# Aligning Words With What Matters: Evaluation of a Nationwide Sample of Pediatric Kidney Transplant Educational Materials

**DOI:** 10.1111/petr.70421

**Published:** 2026-08-03

**Authors:** Taylor R. House, Makayla A. Thomas, Aaron Wightman, Kelly Helm, Ryan J. Coller, Ari Pollack, Susan P. Y. Wong

**Affiliations:** ^1^ Department of Pediatrics University of Wisconsin‐Madison School of Medicine and Public Health Madison Wisconsin USA; ^2^ Department of Pediatrics University of Washington Seattle Washington USA; ^3^ Seattle Children's Hospital Seattle Washington USA; ^4^ Travere Therapeutics San Diego California USA; ^5^ Department of Medicine University of Washington Seattle Washington USA; ^6^ VA Puget Sound Health Care System Seattle Washington USA

**Keywords:** care engagement, educational materials, life participation, patient education, pediatric kidney transplant

## Abstract

**Background:**

Kidney transplant education promotes early partnership between children, caregivers, and clinicians by sharing information and fostering engagement to maximize outcomes. We evaluated educational materials' accessibility, alignment with shared priorities, and portrayal of life participation.

**Methods:**

We performed a content analysis of pediatric kidney transplant centers' educational materials to assess how they addressed 4 key priorities: kidney function, infection, survival, and life participation. We assessed printed materials' readability using Simple Measures of Gobbledygook and Flesch–Kincaid tools and audiovisual materials' understandability and actionability using the Patient Education Materials Assessment Tool.

**Results:**

Of 73 centers approached, 23 (32% response) provided educational packets. Only 6 packets (26%) included materials targeted to children. Average reading grade level was high (SMOG 8.9 ± 1.7; Flesch–Kincaid 9.5 ± 1.8), with only 1 packet written at sixth‐grade level. Audiovisual materials were not highly understandable or actionable. Among key priorities shared by children, caregivers, and clinicians, packets most frequently addressed infection, followed by kidney function, life participation, and survival. We identified 3 themes reflecting portrayal of the key priority of life participation: hope and caution—a proactive, optimistic tone about certain aspects of life participation and cautious tempering of expectations for others; maximizing physical transplant benefits—detailed guidance on physical activity, but limited attention to psychosocial well‐being; and being “normal”—a focus on restoring physical health while framing mental and emotional distress as potential threats to normalcy.

**Conclusions:**

There are important opportunities to enhance transplant educational materials by tailoring content to children, increasing accessibility, and more consistently and comprehensively addressing concerns about life participation after transplant.

## Graphical abstract

Kidney transplant education promotes partnership between children, caregivers, and clinicians by sharing information and fostering engagement to maximize outcomes. This mixed methods analysis of pediatric kidney transplant centers' educational materials demonstrated opportunities to enhance materials by tailoring content to children, increasing accessibility, and more consistently addressing life participation after transplant.

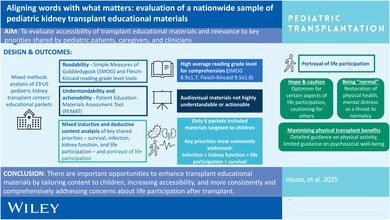

**Abbreviations**
CKD: chronic kidney disease
NAPRTCS: North American Pediatric Renal Trials and Collaborative Studies
PEMAT: Patient Education Materials Assessment Tool
SMOG: Simple Measures of Gobbledygook
SONG‐Kids: Standardized Outcomes in Nephrology—Children and Adolescents

## 1 Introduction

Kidney transplant is the ideal treatment for children with kidney failure [1]. Compared to dialysis, kidney transplant is associated with less morbidity and mortality and greater growth, quality of life, and neuropsychological development [1, 2, 3, 4, 5]. Yet, to fully realize these health benefits, limit the burdens associated with kidney transplant, and achieve the best possible outcomes, children with chronic kidney disease (CKD) and their caregivers must be engaged in care [6, 7].

Engagement encompasses the attitudes and behaviors necessary for patients and caregivers to actively participate in care and address personal priorities to improve outcomes [8, 9]. Successful engagement depends on fostering mutually beneficial partnerships between children, caregivers, and clinicians that are grounded in shared priorities [8, 9]. The Standardized Outcomes in Nephrology—Children and Adolescents (SONG‐Kids) Initiative was an international, multi‐phase initiative that aimed to identify shared priorities in CKD care among children, caregivers, and clinicians. SONG‐Kids identified four core mutual priorities: maximizing survival, preserving kidney function, minimizing infections, and enhancing life participation—a child's ability to meaningfully engage in activities across various areas of life including “medical, lifestyle, mental health, social functioning, independence, and development” [10].

A key opportunity to align care with shared priorities and promote care engagement is by providing children and caregivers with educational materials in preparation for kidney transplant. These materials serve as an important and early point of contact between kidney transplant teams, children, and caregivers, offering a chance to share essential information, develop partnerships, and foster patient and caregiver engagement [11]. Yet, evidence from studies of adults with CKD and kidney transplant recipients suggests that current educational materials are not being fully leveraged toward this end. For example, only 5% of educational materials for adults with CKD meet recommended reading levels [12]. Transplant educational materials often lack tailored information reflecting issues important to adult recipients, such as treatment options, cost, and prognosis [13]. Further, adult kidney transplant recipients report receiving insufficient information about the transplant process, hindering their ability to effectively manage their health [14]. Similar studies in pediatrics found that gaining knowledge is a key coping strategy during the transplant experience [15], and solid organ transplant recipients want more information than they receive [16].

We sought to evaluate the accessibility of pediatric kidney transplant educational materials and their relevance to key priorities shared by pediatric patients, caregivers, and clinicians [10]. Additionally, we assessed how life participation is represented in these materials.

## 2 Methods

### 2.1 Study Design

We performed a mixed methods analysis of transplant educational materials utilized by pediatric kidney transplant centers. The University of Wisconsin‐Madison institutional review board determined this study to be exempt from review. To ensure a comprehensive analysis, our study team comprised experts representing a breadth of disciplines including transplant nephrology, bioethics, palliative care, and family‐centered care, as well as a patient advocate and caregiver with lived experience.

### 2.2 Data Collection

We contacted pediatric kidney transplant programs in the United States associated with the North American Pediatric Renal Trials and Collaborative Studies (NAPRTCS) research consortium between October 2023 and March 2024 to electronically obtain a packet of all English‐language transplant educational materials that they provide to pediatric patients and their caregivers. Programs were contacted via the NAPRTCS email distribution list, and a member of our study team (TRH) also promoted the study during a NAPRTCS meeting. We categorized transplant centers by region and as small (< 10 transplants), medium (10–19 transplants), or large (> 20 transplants) based on the average number of annual transplants performed in the past 2 years [17].

Educational materials from each center were de‐identified prior to analysis. We recorded the number and type of materials used (printed materials, slide decks, web pages, and online modules), the total number of pages comprising written materials, the total number of slides if presentations were provided, the total diagrams used, whether packets included documents provided by the United Network for Organ Sharing (UNOS), and whether materials were targeted for pediatric patients. We excluded consent documents, materials targeted to potential kidney donors, and materials presented in a language other than English.

### 2.3 Readability, Understandability, and Actionability

To estimate the US reading grade level required to comprehend a text, we used two computer‐based readability tools to evaluate written information: Simple Measures of Gobbledygook (SMOG) and Flesch–Kincaid Grade Level [12, 18]. Text from packets were copied into the Sydney Health Literacy Lab's health literacy editor and Microsoft Word (Microsoft Office 365, version 16.89.1) for SMOG and Flesch–Kincaid assessments, respectively [18]. The SMOG formula considers the number of sentences in a text and the number of words with > 2 syllables in 10 sentences near the beginning, middle, and end of the document [18]. The Flesch–Kincaid tool is based on the average number of words per sentence and average number of syllables per word in a document [12]. These formulas have been widely used to evaluate written health information and estimate reading grade level [12, 19, 20].

Two authors (T.R.H. and M.A.T.) rated the understandability and actionability of audiovisual educational materials using the Patient Education Materials Assessment Tool (PEMAT) for Audiovisual Materials [21, 22]. The PEMAT tool includes 17 items with subscales for understandability and actionability. Understandability is based on content, word choice, organization, layout, design, and use of visual aids. Actionability is based on four items—identifying an action the user can take, addressing the user directly when describing actions, breaking down an action into explicit steps, and explaining how to use graphs, tables, charts, or diagrams to take actions. A PEMAT score of > 70% indicates materials are understandable and actionable [22].

### 2.4 Content Analysis

We used a combination of deductive and inductive approaches to content analysis to analyze educational materials [23]. First, TRH (a pediatric nephrologist with advanced qualitative methods training) developed a preliminary codebook of a priori codes based on SONG‐Kids core care priorities—survival, kidney function, infection, and life participation (Box 1) [10, 23]. Next, T.R.H. and M.A.T. (a research coordinator trained in qualitative methods) independently reviewed one packet of educational materials, using the codebook to assign codes to themes identified (deductive coding) as well as openly (inductive) coding for emergent themes related to life participation and how life participation was framed to readers. T.R.H. and M.A.T. then met to review and refine the codes and their definitions, utilizing a consensus‐based approach to resolve any discrepancies [23]. Interrater reliability > 80% was achieved after repeating this process for three additional packets. Either T.R.H. or M.A.T. then used this final codebook to code the remaining packets.

BOX 1 **Core care priorities codes, definitions, and sample quotes.**

**Table:** 

Survival—Discussion of living as long as possible including behaviors that take a person further from, rather than toward, death
Patient survival is usually reported out at the one year and five‐year mark from transplant. There are several reasons as to why transplant patients pass away post‐transplant….
For all ages, transplant survival rates are at least two times better than dialysis.
A transplant is now one of the best treatments for end‐stage kidney disease in children. New drugs and ways of doing surgery have greatly improved the patient survival rate.
Kidney Function—Discussion of how well a kidney is working or functioning including evidence of function in lab values or external signs such as growth or blood pressure
It can take time—a few weeks to a month—before your new kidney works normally.
Immunosuppressants must be taken as long as you want your kidney to function.
When the body recognizes that the kidney is foreign, it sends out white blood cells to attack. The result is kidney damage, eventual dysfunction, and end stage renal failure.
Infection—Discussion of the body being invaded by a microorganism (such as a virus, bacteria, fungus, or parasite) at or after the time of kidney transplant
Besides rejection, infection can be a real danger because your child is taking drugs that block the body's defense system.
Infections that the body can normally fight may take hold and could seriously threaten your child and/or the kidney.
Infections most often occur during the first 3 months after a kidney transplant when your doses of anti‐rejection medicine are highest.
Life participation—Discussion of being able to meaningfully engage in multidimensional aspects of life including “medical, lifestyle, mental health, social functioning, independence, and development” [10].
Now that you've accomplished so much, you may feel like trying new activities you never did before. Go for it!
Post‐traumatic stress disorder and depression can occur as a result of life changes associated with receiving a kidney transplant.
It is important to avoid a direct hit to the new kidney. No contact sports.

## 3 Results

Of the 73 centers approached, 23 (32% response) provided educational packets for analysis (Table 1). On average, one packet included 4 ± 4 documents, a total of 70 ± 51 total pages, and 4 ± 3 diagrams (±SD). Diagrams were primarily used to illustrate anatomy. Over one quarter (30%) of packets included audiovisual presentations. Six packets (26%) included materials targeted to children (47 ± 46 pages), and the intended developmental audience for these materials was not clearly defined.

**TABLE 1:** Characteristics of NAPRTCS‐affiliated centers contributing educational packets.

	*N* = 23
Center size, *n (%)*	
Small, < 10 transplants annually	11 (48)
Medium, 10–19 transplants annually	7 (30)
Large, ≥ 20 transplants annually	5 (22)
Center location, *n (%)*	
Northeast	5 (22)
South	4 (17)
Midwest	8 (35)
West	6 (26)
UNOS documents included, *n (%)*	6 (26)
Materials targeted to child, *n (%)*	6 (26)
Material type included, *n (%)* ^a^	
PDF guidebook/brochure	18 (78)
Online module	2 (9)
Audiovisual presentation	6 (26)
Center fact sheets	5 (22)

Abbreviation: UNOS, United Network for Organ Sharing.

^a^ Totals add to > 100% due to multiple material types at some centers.

### 3.1 Readability, Understandability, and Actionability

The estimated reading grade level required to comprehend educational packet contents was 8.9 ± 1.7 using the SMOG and 9.5 ± 1.8 using Flesch–Kincaid. Only one center offered materials written at sixth grade level. Mean PEMAT scores for audiovisual materials were 70% ± 9% and 69% ± 24% for understandability and actionability, respectively. Despite the widespread use of medical terminology in packets, fewer than half of centers (39%) included a glossary. Several medical terms were used in multiple centers' packets without being defined (Box 2).

BOX 2 **Medical terminology undefined in all education packets.**

**Table:** 

Airborne Cardiovascular Cardiovascular collapse Cauterize Chromosome Conception Contagious Dietary Enteral Exit site Glaucoma	Hickman Immunocompetent Infusion Intervention Intravascular Motor reflex Nausea Organism Parathyroid hormone Pathogen Progressive	Protocol Refractory Regimen Sputum Stroke Tolerance Transmissible Vector Vegetative

### 3.2 References to Key Priorities

Of the four key priorities—kidney function, infection, life participation, and survival—shared by children, caregivers, and clinicians, all packets addressed kidney function and infection, 22 (96%) addressed life participation, and 18 (78%) addressed survival. The terms “infection” appeared 39 ± 38 times per packet; “kidney function” 9 ± 7 times; and “survival” 4 ± 4 times. The term “life participation” did not appear in any packets. Packets included an average of 12 ± 8 pages on infection, 10 ± 6 pages on kidney function, 8 ± 7 pages on life participation, and 3 ± 4 pages on survival.

### 3.3 Elements of Life Participation

References to the key priority of life participation included discussion of participation in school, sports, work, travel, and religious activities; engaging in relationships with romantic partners, family, friends, and pets; and self‐image and psychological well‐being (Table 2). The most common references to life participation were participation in sports and school and psychological well‐being. Other references to life participation were present in fewer than half of all packets, with < 1 page per packet addressing them.

**TABLE 2:** Frequency of elements of life participation addressed in packets.

Element of life participation	# of packets^a^ (%)	Mean # pages per packet (SD)
Sports	19 (83%)	1.5 ± 1.3
Psychological well‐being	17 (74%)	2.1 ± 2.6
School	17 (74%)	2.0 ± 1.8
Romantic relationships	10 (43%)	0.7 ± 0.9
Work	8 (35%)	0.6 ± 1.1
Family	8 (35%)	0.6 ± 1.1
Travel	8 (35%)	0.6 ± 1.1
Religious activities	8 (35%)	0.3 ± 0.5
Self‐image	7 (30%)	0.7 ± 1.9
Friends	4 (17%)	0.4 ± 1.1
Pets	1 (4%)	0.04 ± 0.2

^a^ *N* = 23 packets total.

### 3.4 Framing of Life Participation

We identified three major themes reflecting how life participation was framed in educational packets: hope and caution, maximizing physical transplant benefits, and being “normal.”

#### 3.4.1 Hope and Caution

Portrayal of elements of life participation in packets varied widely, ranging from hopeful enthusiasm to cautious tempering of expectations. Hopeful references encouraged a proactive, optimistic approach to life after transplant, emphasizing not letting the transplant “hold you back.” For example, one packet encouraged: “Now that you've accomplished so much, you may feel like trying new activities you never did before. Go for it! Learn to play an instrument, take karate lessons, try out for a school play.” Packets tended to convey improvements in participation in school, sports, and work after transplant. For example, one document included quotes from prior transplant recipients, with one saying, “Before my transplant, I just sat during recess because I was too uncomfortable to move a lot. Now I'm playing and running around with the rest of the kids for the first time.”

At the same time, packets also cautioned readers to limit expectations about or discouraged other aspects of life after transplant. One packet warned of weight gain and its effects on appearance; “You may feel hungry all the time. Watch out! If you eat the wrong foods, you could gain too much weight. Fat may settle around your belly and upper back or neck, making you look slightly hunched.” Packets tended to convey the risk of deterioration in psychological well‐being, self‐image, and relationships with romantic partners, family, or friends and presented these as potential pitfalls of transplant. For example: “Transplant can be a stressful process. It may put you at risk for depression, post‐traumatic stress disorder, generalized anxiety, anxiety regarding dependence on others, and feelings of guilt.” Another packet read: “If your appearance has changed due to medicines (your face is rounder or you have excess hair), you may feel anxious about what classmates will think.”

#### 3.4.2 Maximizing Physical Transplant Benefits

Packets often highlighted actions patients and caregivers could take or resources they could access through the transplant team to improve physical function after transplant. For example, one packet noted, “Daily play activity, such as walking, running, and riding a bike or big wheel will help your child develop and get stronger. If you have any questions or concerns about your child's progress, talk with your child's regular doctor or someone from the transplant team.” In contrast, packets offered limited guidance on improving psychosocial well‐being or leveraging resources to maximize benefits in these areas after transplant. For instance: “Avoid unhealthy outlets of stress such as abusing drugs or alcohol, overeating, or isolation from friends and family.” Although packets listed adverse psychological outcomes associated with transplant, such as “fear, anxiety, bad dreams, depression, guilt about the donor,” limited guidance beyond, “Emotional support is available should you experience [these],” was explicitly provided on how to address these outcomes.

#### 3.4.3 Being “Normal”

Nearly all packets included mentions of the opportunity to achieve a “normal and healthy” life after transplant. The stated goal of transplant was often to “be normal,” “lead a completely normal life,” or “return to normal,” both for the child and their caregivers. Yet, mental and emotional health were almost always discussed as impediments to “feeling normal.” Patients and caregivers were told to expect many “negative emotions” to accompany transplant including frustration, grief, resentment, injustice, anxiety, uncertainty, and emotional turbulence that could hinder them from “feeling normal.” One packet noted, “A depressed mood or anxiety can prevent your child from leading a normal life.” “Poor mental health” was presented as a “risk” to transplant that could manifest as major depressive disorder, generalized anxiety disorder, and post‐traumatic stress syndrome. No packets explicitly described any potential positive impacts on mental and emotional health following transplant and only described this possibility in vague terms: “Your child may be ready to feel better overall.”

## 4 Discussion

Kidney transplant educational packets used by pediatric centers vary widely in accessibility, content, and emphasis. There were notable gaps in information regarding what it means to participate fully in life after transplant. For children with kidney failure, transplant can be a pivotal moment that brings new possibilities and challenges. Care engagement and aligning care with the priorities of patients and caregivers are essential to helping them navigate this critical time. Our findings highlight significant opportunities to improve transplant educational packets to better support this process.

Educational packets were primarily targeted to caregivers, often containing lengthy documents with medical jargon and exceeding the NIH‐recommended sixth grade reading level for health information for general audiences [20]. Though inclusion of a glossary can support understanding of complex medical concepts [24, 25], fewer than half of educational packets included a glossary. Collectively, these materials may be challenging for many caregivers to utilize—and even less accessible for children. Pediatric transplant recipients and their caregivers want more information about transplant than they currently receive [16]. To be of benefit, educational materials must be developed at a level accessible to the intended audience.

Only six centers' packets included materials targeted to children. Pediatric transplant recipients, particularly adolescents and young adults, are expected to take a major role in self‐management and actively engage in care. They need educational materials tailored to their unique needs. However, patient and caregiver preferences regarding transplant educational packets are largely unknown. Future research should utilize a multi‐site, user‐centered design process to cocreate educational materials with children and caregivers. In addition to developing accessible content, next steps must explore key factors desired by patients and caregivers from diverse developmental, cultural, and educational backgrounds including preferred presentation style, format, modality, timing, and setting for educational packets. This collaborative approach ensures design of a transplant educational packet that is relevant, responsive to patient and caregiver needs, promotes care engagement, and supports broad implementation across transplant centers serving diverse communities [26, 27, 28].

In addition to meeting the needs of patients representing a range of ages, developmental stages, and educational levels, transplant educational packets must also address the topics that matter most to pediatric patients and caregivers. Children, caregivers, and clinicians consistently prioritize minimizing infections, preserving kidney function, maximizing survival, and optimizing life participation [10, 29]. While the production date of packets analyzed is unknown, our findings suggest that current materials may be outdated or misaligned with these priorities. For instance, survival was mentioned on far fewer pages of packets analyzed than other priorities, despite its importance to families. While limited mention of survival may be understandable given the generally favorable survival rates of pediatric kidney transplant recipients, this information gap is consistent with reports from children and their caregivers that this topic is rarely discussed as part of their care [30]. One important factor limiting the inclusion of survival in both educational materials and discussions in clinical care may be nephrologists' discomfort with estimating and sharing prognosis [31]. Addressing such barriers is essential, particularly in light of findings from other populations with serious illness in which adolescents, young adults, and their caregivers have expressed a strong desire not only for prognostic information but also opportunities to engage in advance care planning [32, 33, 34, 35]. Investigating whether young people with CKD hold similar preferences could inform development of transplant educational packets that more effectively address survival and include developmentally appropriate options for advance care planning.

Life participation was addressed infrequently and unevenly across educational packets. While components like participation in school and sports and psychological well‐being were commonly referenced, other important areas, such as peer relationships, religion, and travel appeared in fewer than half of educational packets. Other aspects of life participation important to children and caregivers may have been omitted entirely. This underscores two key issues. First, life participation remains a complex but nebulous concept, and a validated measure of life participation is needed to ensure that it is systematically assessed and addressed [36]. Second, given that educational materials may reflect a limited view of life participation and children and caregivers likely define it in diverse ways, pediatric nephrologists need access to high‐quality serious illness communication training that equips them to elicit each child's and caregiver's unique definition of life participation [31], especially when these priorities may not be fully captured by standardized metrics.

Although a kidney transplant changes the experience of living with CKD, it does not eliminate the barriers to life participation associated with CKD and may even introduce different ones [37]. Aspects of life participation associated with physical function tended to be portrayed optimistically in educational materials, with the expectation of improvement in these areas after transplant. In contrast, psychosocial function [38]—such as psychological well‐being, social roles and interactions, and self‐image—was frequently framed as likely to decline, threatening a sense of normalcy. This framing is inconsistent with existing research showing mixed outcomes in psychosocial domains after transplant. For example, Splinter et al. [39] found no differences in emotional function in pediatric kidney transplant recipients compared to healthy peers, while Anthony et al. [40] reported better emotional functioning after transplant. Similarly, Wightman et al. reported significantly less worry after transplant than before [5]. Casting a negative outlook on psychosocial well‐being after transplant may limit opportunities for post‐traumatic growth—positive psychological change following an extremely challenging life event—which is associated with better kidney function and health‐related quality of life [41]. A more balanced presentation of life participation after transplant could help patients and caregivers develop more realistic expectations and cultivate hope about their transplant journey.

Educational packets often presented transplant as a return to “normal” while also emphasizing numerous psychosocial “risks” that could prevent children from achieving a sense of normalcy. A desire for normalcy is commonly voiced by children with CKD and caregivers [10, 30, 42]. However, this framing in educational material implicitly defines “normal” in narrow terms, presenting emotions like grief, fear, or uncertainty as deviations, and thus, abnormal rather than natural responses after a major health event. Such messaging can exclude diverse experiences and create expectations of how a transplant recipient should feel or act [30, 43]. Rather, children and families should be empowered to determine and share what “normal” means to them, recognizing that these concepts are personal, evolving, and may or may not align with clinicians' perceptions [30, 42, 44]. Flourishing—a state in which all aspects of life are good—may be a more meaningful goal than normalcy [45, 46, 47]. Integrating palliative care early in the transplant journey may support this broader vision by providing a holistic approach to improving quality of life on terms defined by children and their caregivers [48, 49].

This study has some limitations. First, these findings may not be generalizable to pediatric transplant centers that did not provide packets for this study. Though our sample was geographically diverse across centers with variable transplant volumes and reflects a response rate similar to that reported in a comparable NAPRTCs study [50], educational materials were provided by a minority of transplant centers and may not be representative of all programs. Second, though we were able to obtain Powerpoint presentation slides provided to children and caregivers and these provide insights into the standardized information shared with families, we did not have access to the accompanying verbal explanations. Third, our findings represent only packets written in English. The experiences of children and caregivers receiving similar education in other languages warrant future study. Fourth, readability formulas do not measure comprehension or reading ease and may over‐ or underestimate reading levels [51]. The PEMAT is also partially based on subjective assessment of audiovisual educational materials such as whether materials use “common everyday language” and are presented in a “logical sequence” [22]. To mitigate biased assessment of these materials, we used multiple, complementary assessment tools and objective measures. Additionally, while we assumed the clinical accuracy of materials provided by established transplant centers, our analysis utilized measures designed for readability, understandability, and actionability. Evaluating the accuracy of educational content is an important gap for future research. Finally, we analyzed educational packets using established priorities shared among children, caregivers, and clinicians to characterize the current state; but in order for future work to enact necessary, effective changes, the perspectives of pediatric kidney transplant recipients and caregivers must be included.

In summary, we identified important opportunities to improve pediatric kidney transplant educational packets, including increasing their accessibility, tailoring their content to children's developmental needs, and addressing priorities shared by children, caregivers, and clinicians in a more consistent and balanced way. Actively involving children and caregivers in the development process is essential to ensuring that materials align with patients' and caregivers' needs and preferences and promote their engagement from the outset of their transplant journey. Ultimately, a collaborative, holistic approach to education is crucial for moving beyond the goal of achieving “normal” lives for patients and caregivers and toward one aimed at supporting flourishing.

## Funding

Dr. Wong reports receiving research funding from the Department of Veterans Affairs, United States‐United Kingdom Fulbright Commission, and Doris Duke Foundation, which did not pose a financial conflict of interest with respect to this work.

## Conflicts of Interest

All authors declare no conflicts of interest.

## Data Availability

The data that support the findings of this study are available on request from the corresponding author. The data are not publicly available due to privacy or ethical restrictions.

## References

[1] D. L. Gillen , C. O. Stehman‐Breen , J. M. Smith , et al., “Survival Advantage of Pediatric Recipients of a First Kidney Transplant Among Children Awaiting Kidney Transplantation,” American Journal of Transplantation 8, no. 12 (2008): 2600–2606, 10.1111/j.1600-6143.2008.02410.x. 18808405

[2] C. Holmberg and H. Jalanko , “Long‐Term Effects of Paediatric Kidney Transplantation,” Nature Reviews. Nephrology 12, no. 5 (2016): 301–311, 10.1038/nrneph.2015.197. 26656457

[3] A. Francis , M. S. Didsbury , A. van Zwieten , et al., “Quality of Life of Children and Adolescents With Chronic Kidney Disease: A Cross‐Sectional Study,” Archives of Disease in Childhood 104, no. 2 (2019): 134–140, 10.1136/archdischild-2018-314934. 30018070

[4] N. Buyan , M. A. Türkmen , I. Bilge , et al., “Quality of Life in Children With Chronic Kidney Disease (With Child and Parent Assessments),” Pediatric Nephrology 25, no. 8 (2010): 1487–1496, 10.1007/s00467-010-1486-1. 20383649

[5] A. Wightman , M. C. Bradford , and J. Smith , “Health‐Related Quality of Life Changes Following Renal Transplantation in Children,” Pediatric Transplantation 23, no. 2 (2019): e13333, 10.1111/petr.13333. 30548760 PMC10762692

[6] E. K. Hui and S. K. Tsang , “Self‐Determination as a Psychological and Positive Youth Development Construct,” Scientific World Journal 2012 (2012): 759358, 10.1100/2012/759358. 22649322 PMC3353314

[7] H. Aoto , H. Nakatani , S. Kanayama , S. I. Okada , M. Fukada , and K. Hanaki , “Qualitative Analysis of the Psychosocial Adaptation Process in Children With Chronic Kidney Disease: Toward Effective Support During Transition From Childhood to Adulthood,” Yonago Acta Medica 61, no. 3 (2018): 166–174. 30275747 10.33160/yam.2018.09.004 PMC6158356

[8] T. R. House , K. Helm , and A. Wightman , “Building Partnerships to Improve Health Outcomes: Pediatric Patient and Family Engagement in Nephrology Practice,” Advances in Kidney Disease and Health 31, no. 1 (2024): 37–45, 10.1053/j.akdh.2023.10.003. 38403392

[9] T. A. Jerofke‐Owen , N. S. McAndrew , K. S. Gralton , et al., “Engagement of Families in the Care of Hospitalized Pediatric Patients: A Scoping Review,” Journal of Family Nursing 28, no. 2 (2022): 151–171, 10.1177/10748407211048894. 34605283

[10] C. S. Hanson , J. C. Craig , C. Logeman , et al., “Establishing Core Outcome Domains in Pediatric Kidney Disease: Report of the Standardized Outcomes in Nephrology‐Children and Adolescents (SONG‐KIDS) Consensus Workshops,” Kidney International 98, no. 3 (2020): 553–565, 10.1016/j.kint.2020.05.054. 32628942

[11] P. Singer , “Post‐Transplant Education for Kidney Recipients and Their Caregivers,” Pediatric Nephrology 38, no. 7 (2023): 2033–2042, 10.1007/s00467-022-05744-6. 36227432

[12] S. Morony , M. Flynn , K. J. McCaffery , J. Jansen , and A. C. Webster , “Readability of Written Materials for CKD Patients: A Systematic Review,” American Journal of Kidney Diseases 65, no. 6 (2015): 842–850, 10.1053/j.ajkd.2014.11.025. 25661679

[13] S. Koch‐Weser , T. Porteny , D. E. Rifkin , et al., “Patient Education for Kidney Failure Treatment: A Mixed‐Methods Study,” American Journal of Kidney Diseases 78, no. 5 (2021): 690–699, 10.1053/j.ajkd.2021.02.334. 33894282

[14] A. Ghadami , R. Memarian , E. Mohamadi , and S. Abdoli , “Patients' Experiences From Their Received Education About the Process of Kidney Transplant: A Qualitative Study,” Iranian Journal of Nursing and Midwifery Research 17, no. 2 Suppl 1 (2012): S157–S164. 23833599 PMC3696978

[15] M. Korus , J. N. Stinson , R. Pool , A. Williams , and S. Kagan , “Exploring the Information Needs of Adolescents and Their Parents Throughout the Kidney Transplant Continuum,” Progress in Transplantation 21, no. 1 (2011): 53–60, 10.1177/152692481102100107. 21485943

[16] A. Burghall , M. Ruhl , N. Rosaasen , et al., “A Scoping Review of Pediatric Transplant Education,” Pediatric Transplantation 27, no. 7 (2023): e14578, 10.1111/petr.14578. 37528694

[17] Scientific Registry of Transplant Recipients , “Transplant Center Search Results,” accessed January 5, 2024, https://www.srtr.org/transplant‐centers/?query=&distance=50&location=&state=&recipientType=pediatric&organ=kidney&sort=transplantRate.

[18] J. Ayre , C. Bonner , D. M. Muscat , et al., “Multiple Automated Health Literacy Assessments of Written Health Information: Development of the SHeLL (Sydney Health Literacy Lab) Health Literacy Editor v1,” JMIR Formative Research 7 (2023): e40645, 10.2196/40645. 36787164 PMC9975914

[19] J. M. Barnacoat , J. Lewis , K. Stewart , S. S. Mohammad , and S. Paget , “Content and Readability of Patient Educational Materials About Neuromodulation for Childhood Movement Disorders,” Disability and Rehabilitation 46 (2024): 1–7, 10.1080/09638288.2024.2397078. 39246137

[20] S. Sathyanarayanan , P. Paidisetty , L. K. Wang , A. Gosman , S. Williams , and W. Chen , “Assessing the Readability of Online English and Spanish Language Patient Education Resources Provided by the American Society of Plastic Surgeons, American Society of Aesthetic Plastic Surgeons, and American Society of Reconstructive Microsurgeons,” Annals of Plastic Surgery 92, no. 3 (2024): 263–266, 10.1097/SAP.0000000000003754. 38320007

[21] S. J. Shoemaker , M. S. Wolf , and C. Brach , “Development of the Patient Education Materials Assessment Tool (PEMAT): A New Measure of Understandability and Actionability for Print and Audiovisual Patient Information,” Patient Education and Counseling 96, no. 3 (2014): 395–403, 10.1016/j.pec.2014.05.027. 24973195 PMC5085258

[22] Agency for Healthcare Research and Quality , “PEMAT Tool for Audiovisual Materials (PEMAT‐A/V),” Rockville, MD, accessed May 1, 2024, https://www.ahrq.gov/health‐literacy/patient‐education/pemat‐av.html.

[23] N. L. Kondracki , N. S. Wellman , and D. R. Amundson , “Content Analysis: Review of Methods and Their Applications in Nutrition Education,” Journal of Nutrition Education and Behavior 34, no. 4 (2002): 224–230, 10.1016/s1499-4046(06)60097-3. 12217266

[24] S. Koch‐Weser , W. Dejong , and R. E. Rudd , “Medical Word Use in Clinical Encounters,” Health Expectations 12, no. 4 (2009): 371–382, 10.1111/j.1369-7625.2009.00555.x. 19709316 PMC5060502

[25] R. E. Rudd , E. K. Zobel , C. H. Fanta , et al., “Asthma: In Plain Language,” Health Promotion Practice 5, no. 3 (2004): 334–340, 10.1177/1524839903257771. 15228789

[26] R. Harte , L. Glynn , A. Rodriguez‐Molinero , et al., “A Human‐Centered Design Methodology to Enhance the Usability, Human Factors, and User Experience of Connected Health Systems: A Three‐Phase Methodology,” JMIR Human Factors 4, no. 1 (2017): e8, 10.2196/humanfactors.5443. 28302594 PMC5374275

[27] A. R. Dopp , K. E. Parisi , S. A. Munson , and A. R. Lyon , “A Glossary of User‐Centered Design Strategies for Implementation Experts,” Translational Behavioral Medicine 9, no. 6 (2019): 1057–1064, 10.1093/tbm/iby119. 30535343

[28] A. R. Lyon and E. J. Bruns , “User‐Centered Redesign of Evidence‐Based Psychosocial Interventions to Enhance Implementation‐Hospitable Soil or Better Seeds?,” JAMA Psychiatry 76, no. 1 (2019): 3–4, 10.1001/jamapsychiatry.2018.3060. 30427985

[29] A. Tong , S. Samuel , M. Zappitelli , et al., “Standardised Outcomes in Nephrology‐Children and Adolescents (SONG‐Kids): A Protocol for Establishing a Core Outcome Set for Children With Chronic Kidney Disease,” Trials 17 (2016): 401, 10.1186/s13063-016-1528-5. 27519274 PMC4982996

[30] C. S. Hanson , T. Gutman , J. C. Craig , et al., “Identifying Important Outcomes for Young People With CKD and Their Caregivers: A Nominal Group Technique Study,” American Journal of Kidney Diseases 74, no. 1 (2019): 82–94, 10.1053/j.ajkd.2018.12.040. 30885704

[31] T. R. House , A. Wightman , J. Smith , M. Schwarze , M. C. Bradford , and A. R. Rosenberg , “Palliative Care Training in Pediatric Nephrology Fellowship: A Cross‐Sectional Survey,” Kidney360 4 (2023): 1437–1444, 10.34067/KID.0000000000000233. 37531201 PMC10615382

[32] T. M. Kazmerski , D. J. Weiner , J. Matisko , et al., “Advance Care Planning in Adolescents With Cystic Fibrosis: A Quality Improvement Project,” Pediatric Pulmonology 51, no. 12 (2016): 1304–1310, 10.1002/ppul.23559. 27749020

[33] S. Z. Bedoya , A. Fry , M. L. Gordon , et al., “Adolescent and Young Adult Initiated Discussions of Advance Care Planning: Family Member, Friend and Health Care Provider Perspectives,” Frontiers in Psychology 13 (2022): 871042, 10.3389/fpsyg.2022.871042. 35756319 PMC9215331

[34] L. Wiener , S. Zadeh , H. Battles , et al., “Allowing Adolescents and Young Adults to Plan Their End‐of‐Life Care,” Pediatrics 130, no. 5 (2012): 897–905, 10.1542/peds.2012-0663. 23045560 PMC3483891

[35] D. D. DeCourcey , M. Silverman , A. Oladunjoye , and J. Wolfe , “Advance Care Planning and Parent‐Reported End‐Of‐Life Outcomes in Children, Adolescents, and Young Adults With Complex Chronic Conditions,” Critical Care Medicine 47, no. 1 (2019): 101–108, 10.1097/CCM.0000000000003472. 30303834

[36] J. Kerklaan , E. Hannan , A. Baumgart , et al., “Patient‐ and Parent Proxy‐Reported Outcome Measures for Life Participation in Children With Chronic Kidney Disease: A Systematic Review,” Nephrology, Dialysis, Transplantation 35, no. 11 (2020): 1924–1937, 10.1093/ndt/gfaa132. 32743664

[37] T. R. House and A. Wightman , “Adding Life to Their Years: The Current State of Pediatric Palliative Care in CKD,” Kidney360 2, no. 6 (2021): 1063–1071, 10.34067/KID.0000282021. 35373080 PMC8791371

[38] N. Altunsoy , D. S. Di Ki Ci , F. P. Cokmus , et al., “Evaluation of Psychosocial Functioning in the Acute Treatment Term of Major Depressive Disorder: A 16‐Week Multi‐Centered Follow‐Up Study,” Asian Journal of Psychiatry 66 (2021): 102883, 10.1016/j.ajp.2021.102883. 34700179

[39] A. Splinter , L. A. Tjaden , L. Haverman , et al., “Children on Dialysis as Well as Renal Transplanted Children Report Severely Impaired Health‐Related Quality of Life,” Quality of Life Research 27, no. 6 (2018): 1445–1454, 10.1007/s11136-018-1789-4. 29374855 PMC5951873

[40] S. J. Anthony , D. Hebert , L. Todd , et al., “Child and Parental Perspectives of Multidimensional Quality of Life Outcomes After Kidney Transplantation,” Pediatric Transplantation 14, no. 2 (2010): 249–256, 10.1111/j.1399-3046.2009.01214.x. 19686446

[41] R. P. Nash , M. M. Loiselle , J. L. Stahl , et al., “Post‐Traumatic Stress Disorder and Post‐Traumatic Growth Following Kidney Transplantation,” Kidney360 3, no. 9 (2022): 1590–1598, 10.34067/KID.0008152021. 36245667 PMC9528379

[42] A. Tong , R. Morton , K. Howard , S. McTaggart , and J. C. Craig , “‘When I Had My Transplant, I Became Normal’: Adolescent Perspectives on Life After Kidney Transplantation,” Pediatric Transplantation 15, no. 3 (2011): 285–293, 10.1111/j.1399-3046.2010.01470.x. 21281416

[43] M. Rost , “‘To Normalize Is to Impose a Requirement on an Existence’: Why Health Professionals Should Think Twice Before Using the Term ‘Normal’ With Patients,” Journal of Bioethical Inquiry 18, no. 3 (2021): 389–394, 10.1007/s11673-021-10122-2. 34661847 PMC8521090

[44] T. Gutman , C. S. Hanson , S. Bernays , et al., “Child and Parental Perspectives on Communication and Decision Making in Pediatric CKD: A Focus Group Study,” American Journal of Kidney Diseases 72, no. 4 (2018): 547–559, 10.1053/j.ajkd.2018.05.005. 29980375

[45] C. Brunson , T. R. House , D. Noone , and A. Wightman , “Management Dilemmas in Pediatric Nephrology: Moving From Friction to Flourishing in ‘Challenging’ Cases,” Pediatric Nephrology 39, no. 11 (2024): 3363–3371, 10.1007/s00467-024-06384-8. 38668777

[46] T. R. House and A. Wightman , “Enabling Flourishing: Novel Approaches in Palliative Medicine for Children With Advanced Kidney Disease,” Current Opinion in Nephrology and Hypertension 32 (2022): 41–48, 10.1097/MNH.0000000000000839. 36250456

[47] A. Wightman , “Articulating the Value of Pediatric Priority and Transplant for Children,” Pediatric Transplantation 27, no. 7 (2023): e14595, 10.1111/petr.14595. 37608472

[48] M. Thomas , L. Mosley , and T. R. House , “Chronic Kidney Disease Takes, Palliative Care Gives: What a Pediatric Nephrologist Needs to Know About Palliative Care,” Current Pediatrics Reports 12 (2024): 185–193, 10.1007/s40124-024-00327-5.

[49] Center to Advance Palliative Care , “About Palliative Care,” https://www.capc.org/about/palliative‐care/.

[50] V. R. Dharnidharka , K. L. Martz , D. M. Stablein , and M. R. Benfield , “Improved Survival With Recent Post‐Transplant Lymphoproliferative Disorder (PTLD) in Children With Kidney Transplants,” American Journal of Transplantation 11, no. 4 (2011): 751–758, 10.1111/j.1600-6143.2011.03470.x. 21446977

[51] Agency for Healthcare Research and Quality , “Tip 6. Use Caution With Readability Formulas for Quality Reports,” accessed October 1, 2024, https://www.ahrq.gov/talkingquality/resources/writing/tip6.html.

